# Draft Genome Sequences of Three Strains of Pseudomonas syringae pv. eriobotryae, a Pathogen Causing Canker Disease in Loquat, Isolated in Japan

**DOI:** 10.1128/MRA.01049-20

**Published:** 2021-01-07

**Authors:** Hiroaki Tashiro, Yukio Nagano, Ayaka Jiromaru, Ryunosuke Sakaguchi, Naofumi Hiehata, Shinji Fukuda

**Affiliations:** a Saga University, Center for Education and Research in Agricultural Innovation, Saga, Japan; b Saga University, Analytical Research Center for Experimental Sciences, Saga, Japan; c Agriculture and Forestry Technical Development Center, Nagasaki Prefectural Government, Omura, Nagasaki, Japan; Loyola University Chicago

## Abstract

The phytopathogenic bacterium Pseudomonas syringae pv. eriobotryae causes canker disease in loquat. Isolates from Japan are classified into three groups based on pathogenicity and pigment production. In this study, we report the draft genome sequences of three strains, one belonging to each of the three groups.

## ANNOUNCEMENT

Loquat (Eriobotrya japonica [Thunb.] Lindl.), a subtropical evergreen fruit tree, is widely cultivated in subtropical regions. Pseudomonas syringae pv. eriobotryae causes loquat canker disease, the most serious disease in loquat cultivation (1). Isolates from Japan are classified into three groups: group A is not pathogenic to mesophyll and produces no pigment in culture medium; group B is pathogenic to mesophyll and produces no pigment; group C is not pathogenic to mesophyll and produces brown pigment (2). In loquat, a single dominant gene (*Pse-a*) controls resistance to group A (3), and a single recessive gene (*Pse-c*) controls resistance to group C (4). Here, we report the draft genome sequences of three strains isolated in Japan (AM001, BM001, and CG001), one from each group.

Three strains of Pseudomonas syringae pv. eriobotryae were isolated from a leaf from a diseased loquat tree (3, 4). The bacteria were cultured at 37°C for 16 to 18 h in King’s medium (5) with shaking. DNA was isolated using cetyltrimethylammonium bromide (CTAB)/NaCl and phenol-chloroform extractions, followed by isopropanol precipitation (6). Sequencing libraries were generated using the NEBNext Ultra DNA library prep kit for Illumina (NEB, USA). The libraries were sequenced with 150-bp paired-end reads by Novogene (Beijing, China), using a NovaSeq 6000 system (Illumina, San Diego, CA, USA). Adapter sequences and low-quality bases were trimmed using Trimmomatic v 0.39 (ILLUMINACLIP:adapter_sequence:2:30:10 LEADING:20 TRAILING:20 SLIDINGWINDOW:5:20 MINLEN:50) (7). Assembly was performed using Unicycler v 0.4.8 (8), and gene annotation was performed using DFAST (https://dfast.nig.ac.jp) (9). To construct a phylogenetic tree, all combinations of 2,246 orthologous proteins were extracted using OrthoFinder v 2.3.12 (-og) (10), multiple alignments were performed using MAFFT v 7.471 (--maxiterate 1000 --localpair) (11) and trimmed using trimAl v 1.4 (-automated1) (12), and a maximum-likelihood tree was constructed using IQ-TREE (-sp, -bb 1000) (v 2.0.6) (13). Default parameters were used for all software tools, unless otherwise stated.

The resulting genome assemblies are shown in Table 1.

**TABLE 1:** Assembly metrics and annotated features of three strains of Pseudomonas syringae pv. eriobotryae isolated in Japan

Strain	BioSample accession no.	Group	No. of reads	Total sequence length (bp)	No. of contigs	Avg coverage (×)	*N*_50_ (bp)	No. of CDSs^*a*^	No. of tRNAs	No. of rRNAs	G+C content (%)	Coding ratio (%)	DDBJ/GenBank accession no.	SRA accession no.
AM001	SAMD00239674	A	8,367,914	6,576,215	226	184	152,814	5,989	54	4	57.6	84.9	BMZW00000000.1	DRA010831
BM001	SAMD00239675	B	9,420,632	6,324,864	140	202	151,127	5,780	77	4	57.6	85.9	BMZX00000000.1	DRA010830
CG001	SAMD00239676	C	8,506,582	6,327,638	227	194	149,087	5,784	55	3	58.0	85.2	BMZY00000000.1	DRA010832

a CDSs, coding DNA sequences.

**FIG 1:**
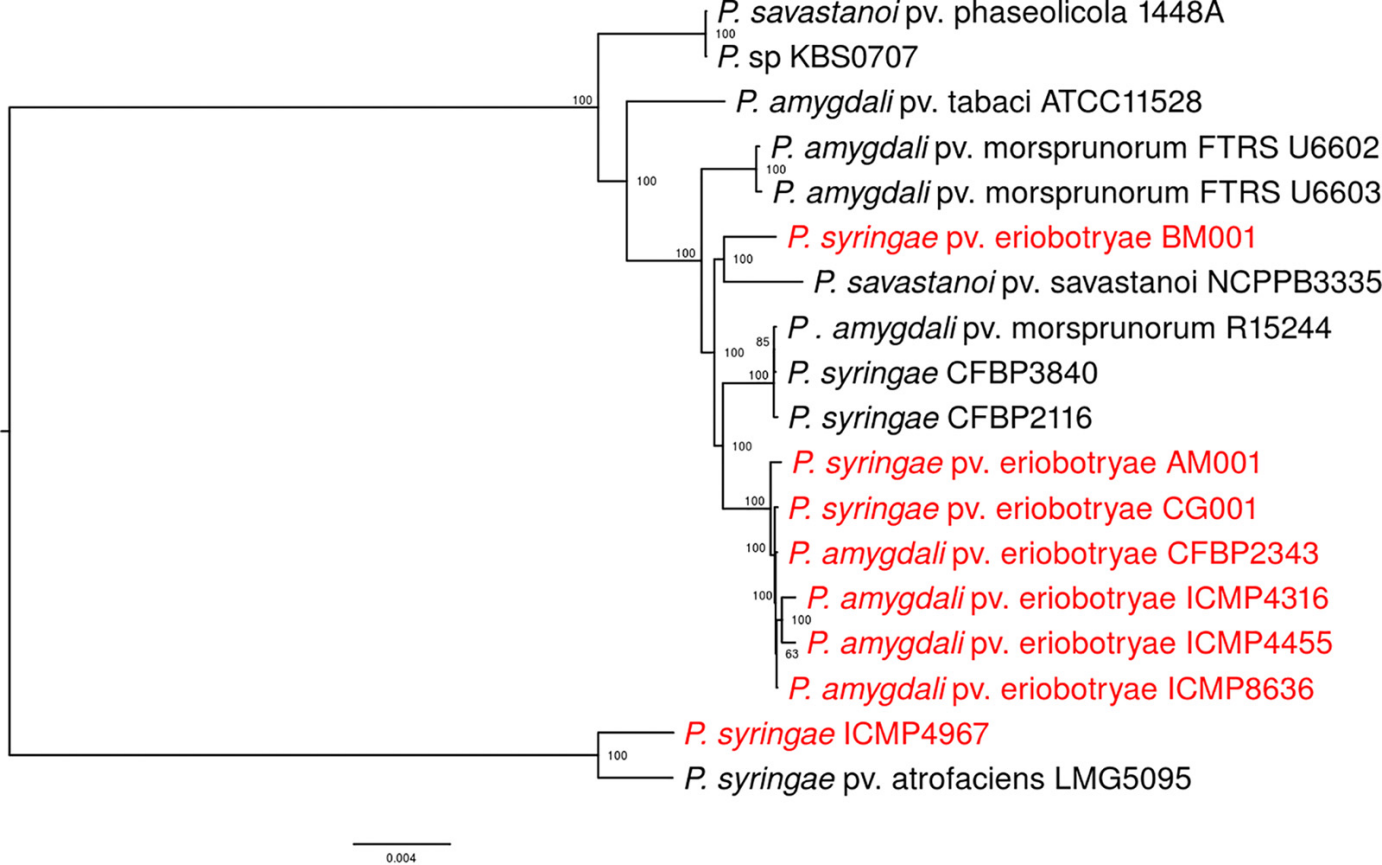
Maximum-likelihood phylogenetic tree, based on all 2,246 orthologous proteins, showing the phylogenetic relationship between P. syringae pv. eriobotryae from Japan and other *Pseudomonas* species. Strains pathogenic to loquat are shown in red. Bootstrap values (%) for 1,000 repetitions are given at the nodes. The strain names and GenBank RefSeq accession numbers are as follows: CFBP2343 (GCF_001538055.1), ICMP4316 (GCF_003700715.1), ICMP4455 (GCF_001400455.1), ICMP4967 (GCF_003700015.1), ICMP8636 (GCF_003699445.1), 1448A (GCF_000012205.1), KBS0707 (GCF_005937945.2), ATCC 11528 (GCF_000145945.2), FTRS U6602 (GCF_002939205.1), FTRS U6603 (GCF_002939245.1), NCPPB3335 (GCF_000164015.3), R15244 (GCF_002905685.2), CFBP3840 (GCF_900235815.1), CFBP2116 (GCF_900289125.1), and LMG5095 (GCF_003047185.1).

The draft genome sequences of five *Pseudomonas* strains pathogenic to loquat (CFBP2343, ICMP4316, ICMP4455, ICMP4967, and ICMP8636) are available in DDBJ/EMBL/GenBank. The source of ICMP8636 is unknown; the other four strains were isolated in the United States. We additionally used the genome sequences of 10 *Pseudomonas* strains pathogenic to the other plants (1448A, KBS0707, ATCC 11528, FTRS U6602, FTRS U6603, NCPPB3335, R15244, CFBP3840, CFBP2116, and LMG5095) to construct a phylogenetic tree (Fig. 1). In this tree, two Japanese strains (AM001 and CG001), three U.S. strains (CFBP2343, ICMP4316, and ICMP4455), and ICMP8636 formed a single clade. Because loquat was introduced into the United States from Japan or China, the three U.S. strains and ICMP8636 probably came from East Asia. Interestingly, one of the Japanese strains, BM001, was closely related to P. savastanoi pv. savastanoi NCPPB3335, the causal agent of olive knot disease. Mechanisms such as horizontal gene transfer and hybridization might have genetically separated BM001 from the other two Japanese strains. More interestingly, one of the U.S. strains, ICMP4967, was genetically distant from the other seven strains. Although further study is required, the progenitor of this strain might have been present in the United States before the introduction of loquat.

The draft genome sequences reported here will provide fundamental information for elucidating the mechanisms of infection of P. syringae pv. eriobotryae and facilitate progress toward protection against this pathogen.

### Data availability.

The draft genome sequences and corresponding read data are available in DDBJ/GenBank. The DDBJ/GenBank and SRA accession numbers for P. syringae pv. eriobotryae AM001, BM001, and CG001 are listed in Table 1.
